# ﻿*Capitojoppa*, a new genus of Ichneumoninae (Hymenoptera, Ichneumonidae) from Peruvian Amazonia

**DOI:** 10.3897/zookeys.1178.108929

**Published:** 2023-09-01

**Authors:** Brandon R. Claridge, Kari M. Kaunisto, Ilari E. Sääksjärvi

**Affiliations:** 1 Utah State University, 5305 Old Main Hill, Logan, UT, 84322 USA Utah State University Logan United States of America; 2 Biodiversity Unit, Zoological Museum, University of Turku, 20014 Turku, Finland University of Turku Turku Finland

**Keywords:** Allpahuayo-Mishana, biodiversity, Neotropical, rainforests, South America, taxonomy, tropical

## Abstract

A new monotypic genus of ichneumonine parasitoid wasps (Hymenoptera, Ichneumonidae, Ichneumoninae) is described from Peru; *Capitojoppa***gen. nov.** is described to accommodate *Capitojoppaamazonica***sp. nov.** The new genus is morphologically very distinctive and can be easily separated from all known ichneumonine genera. By describing *Capitojoppa* from the lowland rain forests of Peru, we aim to draw attention to the considerable diversity and morphological variation of the Amazonian ichneumonine fauna.

## ﻿Introduction

The vast Amazonian rainforest accommodates a species-rich but still very little-known fauna of ichneumonid parasitoid wasps (Hymenoptera). Among the best sampled Amazonian study localities for ichneumonids is the Peruvian National Reserve of Allpahuayo-Mishana (AMNR), located near the city of Iquitos. During the last three decades the last author (IES) has inventoried the ichneumonid fauna of Allpahuayo-Mishana mainly by long-term Malaise trapping (e.g. Sääksjärvi et al. 2004; Veijalainen et al. 2013; Gómez et al. 2018). Besides documenting an unprecedented abundance and diversity of tropical parasitoid wasps, these field studies conducted in this geologically and environmentally heterogenous national reserve have yielded a plethora of unknown ichneumonid taxa, many of them apparently morphologically highly specialized.

Among the most abundant ichneumonid subfamilies in AMNR is Ichneumoninae (Veijalainen et al. 2013). This subfamily is cosmopolitan and usually well represented in all biogeographic regions. However, despite the large size and vivid coloration of many ichneumonine species, the Neotropical fauna of the subfamily remains little known. Heinrich (1977), in his comprehensive revision of Floridan ichneumonines, claimed that tropical lowland rainforests are “always poorly populated by Ichneumoninae”. However, Gauld (1991), after discovering between 300 and 400 species from Costa Rica, reported ichneumonines as abundant in all terrestrial habitats of the country. Finally, Veijalainen et al. (2013), after studying a large amount of canopy fogging and Malaise trap samples collected from Costa Rica, Ecuador, and Peru, showed Ichneumoninae to be among the most abundant subfamilies in Neotropical habitats.

In 2022, we started the identification of AMNR ichneumonines into genera and morphospecies. This work revealed a species-rich fauna of ichneumonines from western Amazonia. We now aim to publish a series of papers on these discoveries. We are beginning this effort with a description of a specialized and morphologically distinctive genus of Ichneumoninae from AMNR. With this article, we are hoping to draw attention to the considerable diversity and morphological variation of the Amazonian ichneumonine fauna.

## ﻿Material and method

Morphological terminology primarily follows Bennett et al. (2019), except the use of “median field” and antennal morphology which follows Heinrich (1960). “T1”, “T2”, etc. refer to the corresponding metasomal tergites. The generic description was informed by Heinrich (1960, 1977) and Townes and Townes (1966), mainly focusing on major structural characters that differ at supraspecific levels. The female holotype is described in full, while only variations are noted for the male paratype.

Both specimens were collected by IES in the National Reserve of Allpahuayo Mishana (NRAM), located about 25 km south-west of the large city of Iquitos, Department of Loreto, Peru. The NRAM lies between 100 and 200 m above sea level in a lowland tropical rain-forest terrain. The NRAM forests are known to be extremely heterogeneous in structure and species composition (Sääksjärvi et al. 2006).

Digital images were taken using a Sony a9 ii digital camera and Mitutoyo microscope lenses. Resulting photos were then stacked with Helicon Focus and post-processed with Adobe Photoshop CC.

The holotype and paratype will be deposited in the Universidad Nacional Mayor de San Marco (**MUSM**).

## ﻿Results

### ﻿ Capitojoppa

Taxon classificationAnimaliaHymenopteraIchneumonidae

Claridge, Sääksjärvi & Kaunisto gen. nov.

46CCB75E-629F-56DC-BA5A-762996FAC673

https://zoobank.org/DCE30183-1262-42CE-A34C-6348D04AF9B3

Figs 1 , 2

#### Type species.

*Capitojoppaamazonica* Claridge, Sääksjärvi & Kaunisto, sp. nov.

#### Diagnosis.

*Capitojoppa* belongs to the tribe Ichneumonini and is diagnosed by the following combination of characters: overall robust body with an enlarged head and a wide gena; large mandibles with widely spaced, subequal teeth; labrum exposed and visible in anterior view; supraclypeal area and clypeus without lateral ridge as in *Joppa* Fabricius and *Projoppa* Townes; occipital carina complete and meeting hypostomal carina at mandibular base; wing membrane clear; propodeum without distinct dorsal and posterior faces (anterior margin strongly sloping and propodeum gently sloping posteriorly); dorsomedial surface of hind coxa coarsely striate; hind coxa without scopa; T1 longitudinally striate; T2 longitudinally striate medially; gastrocoelus deeply impressed; thyridium well developed; and female metasomal apex oxypygous.

Overall, *Capitojoppa* is most similar to *Joppa* due to the following characters: mandibles large with subequal teeth; propodeum lacking distinct dorsal and posterior faces due to both the anterior and posterior margins sloping (variable in *Joppa* but usually as in *Capitojoppa*); gastrocoelus deeply impressed; thyridium well developed; and T1–2 coarsely, longitudinally striate. *Capitojoppa* primarily differs from *Joppa* by the exposed labrum which is visible in anterior view (concealed by the elongated clypeus in *Joppa*); the lack of distinct lateral ridges on the supraclypeal area and clypeus (a defining character in *Joppa*); the coarse, longitudinal striation on the supra-antennal area (smooth and without striation or rugosity in *Joppa*); complete occipital carina (obsolete ventrally in *Joppa*); clear wings (yellow with black markings in *Joppa*); and coarse striation on dorsomedial surface of the hind coxa (smooth and without striation or rugosity in *Joppa*). Also, the habitus is considerably more robust than any *Joppa* species examined.

#### Description.

***Head*.** Mandible large with subequal, widely separated teeth. Clypeus flat and with ventral margin straight. Epistomal suture subobsolete. Malar space ~0.7× basal mandibular width. Clypeus and supraclypeal area simple, without sublateral ridges. Dorsal margin of median field with small medial tubercle. Supra-antennal area coarsely, longitudinally striate. Gena wide. Female flagellum bristle-shaped (flattened and widened past midpoint before acutely tapering apically); basal flagellomeres moderately slender (flagellomere 1 ~3.0× as wide as long). Male flagellum with tyloids present and beginning on flagellomere 10 (endpoint of tyloids unknown); bristle ridges present beginning on flagellomere 11 (development of bristle ridges uncertain due to missing flagellum past flagellomere 15 on male paratype).

***Mesosoma*.** Epomia present. Notaulus obsolete. Scutellum flat, not significantly elevated above postscutellum; not laterally carinate. Sternaulus obsolete. Posterior transverse carinae of the mesothoracic venter only present laterally. Juxtacoxal carina present. Propodeal carinae largely obsolete with only anterior transverse carina well developed medially and longitudinal carinae developed at extreme posterior (carinae more strongly developed in male specimen). Anterior margin of propodeum without medial tubercle. Legs stout. Fore and mid tibiae with well-developed, decumbent, conical spines (more strongly developed in female). Hind coxa without scopa and with dorsomedial surface coarsely striate. Tarsal claws simple, not pectinate.

***Wings*.** Areolet pentagonal. Distal abscissa of 1/Cu present.

***Metasoma*.** Metasoma rather stout, but linear and weakly tapering apically. T1 coarsely, longitudinally striate, median field indistinct. T2 longitudinally striate medially with striate area becoming narrower posteriorly. Thyridium well developed and wide (thyridium ~1.2× interthyridial width). Gastrocoelus deeply impressed. Female metasomal apex oxypygous. Ovipositor not significantly projecting past metasomal apex. Male hypopygium simple, not medially elongated. Gonoforceps simple, not enlarged.

#### Etymology.

Derived from *capito*, meaning big-headed in medieval Latin, combined with the generic name, *Joppa*. In addition, *Capito* refers to the Neotropical bird genus *Capito* (barbets), including species of stocky birds with heavy bills. Gender feminine.

#### Comments.

Sexual dimorphism is minimal with only minor colour and surface sculpture differences between the female and male specimens.

The similarity to *Joppa* and other characters typical of the group (females with bristle-shaped antennae and males with bristle-ridges) suggests that *Capitojoppa* belongs to a clade of Neotropical genera that includes *Carinodes* Hancock, *Joppa*, *Joppocryptus* Viereck, *Limonethe* Townes, *Lobaegis* Townes, *Lophojoppa* Brèthes, *Narthecura* Townes, *Notacma* Townes, *Oezdemirus* Ozdikmen & Turgut, *Ortezia* Cresson, and *Trogomorpha* Ashmead (Santos et al. 2021).

### ﻿ Capitojoppa amazonica

Taxon classificationAnimaliaHymenopteraIchneumonidae

Claridge, Sääksjärvi & Kaunisto sp. nov.

19901E31-19DC-57F6-8CFE-C0F54C9361F4

https://zoobank.org/6DB53656-D931-46FC-8972-D0E063D541A5

Figs 1 , 2

#### Description.

**Figure 1.**
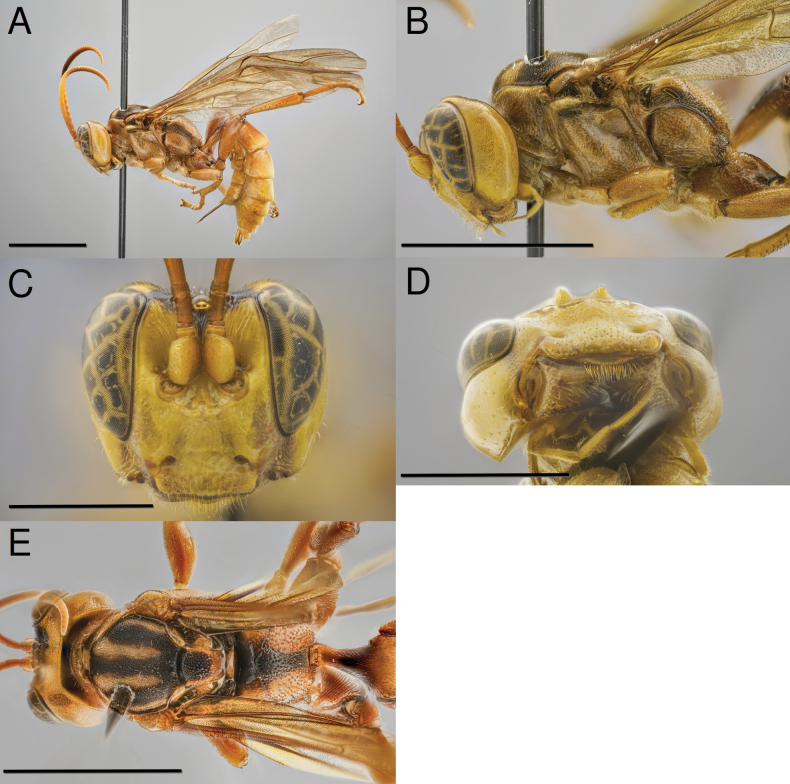
*Capitojoppaamazonica* sp. nov., holotype female **A** habitus **B** mesosoma, lateral view **C** head, anterior view **D** head, ventral view **E** mesosoma, dorsal view. Scale bars: 5 mm (**A, B, E**); 2 mm (**C, D**).

**Female** (Fig. 1A–E). Body length: 17.7 mm.; fore wing length: 14.0 mm.

***Colour*.** Overall yellow or brownish-yellow with restricted black areas. Head yellow except for: black mandibular apex; T-shaped black mark at vertex with narrow, medial extension to antennal bases; small dark brown mark immediately dorsal to antennal sockets; and occiput with dorsal 0.3 dark brown to black. Scape and pedicel brownish-yellow. Female flagellum with flagellomeres 1–5 yellowish-brown, flagellomeres 6–10 brownish-yellow, and the remainder brown ventrally and dark brown dorsally. Pronotum yellow except for small submedial brownish marks in female and small. Propleuron yellow. Mesonotum dark-brown to black except for submedial brownish-yellow stripes and posterior 0.6 with lateral margin brownish-yellow. Scutellum black anteromedially becoming yellow posterolaterally. Mesopleuron yellow except for linear, longitudinal black mark immediately ventral to subalar ridge. Postscutellum yellow. Ventral division of metapleuron yellow except for posteroventral margin dark brown. Propodeum varying from primarily yellow wide and posteriorly tapering black medial longitudinal stripe and lateral margins black to primarily black with only areas corresponding to second lateral areas yellow. Fore and mid legs with coxae and trochanters yellow; femora yellowish-brown; tibiae brownish-yellow except at base; fore tarsus brownish-yellow; and mid tarsus yellowish-brown except for tarsomere 1 brownish-yellow. Hind leg with coxa yellowish ventrally and brownish dorsally except for dark brown at apex; trochanter brown basally with remainder yellowish; femur yellowish-brown; tibia primarily yellowish-brown except for partially yellowish on anterior face; tarsus brown. Metasoma primarily brownish-yellow except for anterior 0.7 of T1 black dorsally.

***Head*.** Clypeus smooth with moderately dense, coarse punctation. Supraclypeal area smooth medially becoming weakly granulate laterally, finely, densely punctate medially becoming sparser laterally. Supra-antennal area smooth and coarsely, longitudinally striate. Gena smooth and impunctate. Vertex smooth and weakly, irregularly sculptured except for small weakly granulate area between lateral ocelli and eye margin.

***Mesosoma*.** Propleuron smooth and impunctate except for a few scattered punctures. Pronotum smooth and finely, nearly indistinctly, punctate at dorsal and posterior margins. Mesonotum densely, finely punctate with punctures adjacent to subadjacent. Scutellum smooth and varying from densely punctate. Mesopleuron smooth and moderately puncate posteroventrally becoming sparser anteriorly and dorsally. Speculum smooth and impunctate. Propodeum predominantly weakly granulate and coarsely punctate with punctures becoming denser posteriorly and laterally, basal area smooth and impunctate. Hind coxa with dorsomedial surface coarsely striate and ventral surface densely punctate with some punctures forming oblique rugulae.

***Metasoma*.** T1 coarsely, longitudinally striate. T2 longitudinally striate medially with striate area becoming narrower posteriorly, remainder densely punctate with punctures subadjacent.

**Figure 2.**
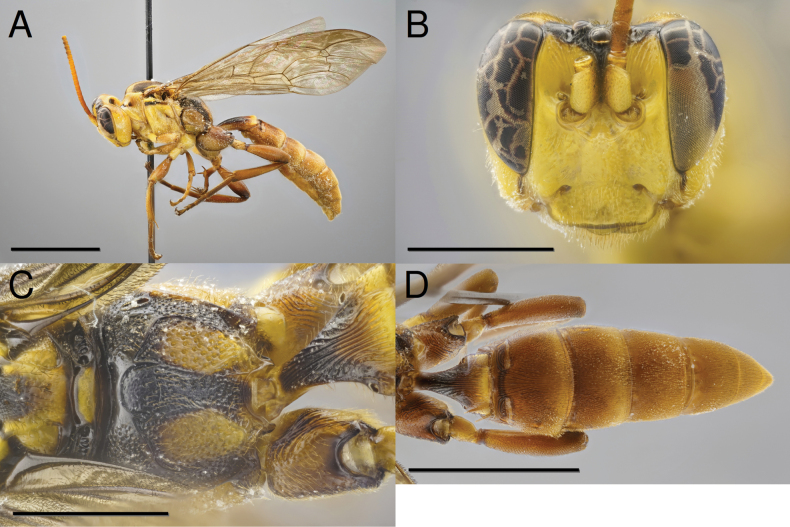
*Capitojoppaamazonica* sp. nov., paratype male **A** habitus **B** head, lateral view **C** propodeum, dorsal view **D** hind coxae and metasoma, dorsal view. Scale bars: 5 mm (**A, D**); 2 mm (**B, C**).

**Male.** (Fig. 2A–D). Body length: 17.0 mm; fore wing length: 13.6 mm. As in female except for: small, additional sublateral brownish marks on propleuron; clypeus coarsely, densely punctate; and scutellum finely, sparsely punctate.

#### Materials examined.

***Holotype*** (female): Peru, Dept. of Loreto, Iquitos area, Mishana, 1–16.12.1998, clay, Ilari E. Sääksjärvi et al. Leg., Malaise trap, APHI A1/8 061 (MUSM). ***Paratype*** (male): Peru, Dept. of Loreto, Iquitos area, Allpahuayo, 31.10–6.11.2011, Gómez & Sääksjärvi leg., Malaise 11, Week24, 30°58'35"S, 73°25'57"W (MUSM).

#### Distribution.

Only known from type locality in western Peruvian Amazon (National Reserve of Allpahuayo-Mishana). Despite examining extensive Neotropical materials in the Biodiversity Unit, Zoological Museum of University of Turku (Finland), the Entomological Museum of Utah State University (USA), and the Natural History Museum of the Alexander Humboldt Institute in Bogotá, (Colombia), especially from Costa Rica, Panama, and montane regions of Colombia, no other specimens of this genus were found. Thus, *Capitojoppa* may be restricted to the lowland Amazon Basin.

#### Biology.

The host(s) or other biological information are unknown.

#### Etymology.

The specific name “*amazonica*” refers to Amazonia, the largest and most diverse rainforest on Earth.

## Supplementary Material

XML Treatment for Capitojoppa

XML Treatment for Capitojoppa amazonica

## ﻿Additional information

### Author ORCIDs

Brandon R. Claridge https://orcid.org/0000-0002-2222-326X

Kari M. Kaunisto https://orcid.org/0000-0001-6665-0047

Ilari E. Sääksjärvi https://orcid.org/0000-0002-8107-5607

### Data availability

All of the data that support the findings of this study are available in the main text.
